# Smartphones and Cognition: A Review of Research Exploring the Links between Mobile Technology Habits and Cognitive Functioning

**DOI:** 10.3389/fpsyg.2017.00605

**Published:** 2017-04-25

**Authors:** Henry H. Wilmer, Lauren E. Sherman, Jason M. Chein

**Affiliations:** Department of Psychology, Temple University, PhiladelphiaPA, USA

**Keywords:** smartphones, mobile technology, media multitasking, attention, memory, delay of gratification and delay discounting, everyday cognition

## Abstract

While smartphones and related mobile technologies are recognized as flexible and powerful tools that, when used prudently, can augment human cognition, there is also a growing perception that habitual involvement with these devices may have a negative and lasting impact on users’ ability to think, remember, pay attention, and regulate emotion. The present review considers an intensifying, though still limited, area of research exploring the potential cognitive impacts of smartphone-related habits, and seeks to determine in which domains of functioning there is accruing evidence of a significant relationship between smartphone technology and cognitive performance, and in which domains the scientific literature is not yet mature enough to endorse any firm conclusions. We focus our review primarily on three facets of cognition that are clearly implicated in public discourse regarding the impacts of mobile technology – attention, memory, and delay of gratification – and then consider evidence regarding the broader relationships between smartphone habits and everyday cognitive functioning. Along the way, we highlight compelling findings, discuss limitations with respect to empirical methodology and interpretation, and offer suggestions for how the field might progress toward a more coherent and robust area of scientific inquiry.

## Introduction

As portable media devices, such as smartphones, have become an increasingly pervasive part of our lives, they have also become increasingly capable of supplementing, or even supplanting, various mental functions. With the capacity to be used as phonebooks, appointment calendars, internet portals, tip calculators, maps, gaming devices, and much more, smartphones seem capable of performing an almost limitless range of cognitive activities for us, and of satisfying many of our affective urges. However, sensationalist articles with titles such as, “Are Smartphones Making Us Dumber?” (Ellison, 2012) and, “Is Your Smartphone Making You Fat and Lazy?” (Morin, 2013) encourage the conclusion that reliance on smartphones and related technologies is not aiding mental functioning, but rather, is having a negative impact on our ability to think, remember, pay attention, and regulate emotion. Some have even made the claim that modern connectedness is “rewiring our brains” to constantly crave instant gratification, and that this threat to our society is “almost as important as climate change” (Greenfield, 2013). Are these simply examples of an older generation once again thinking its “progeny yet more corrupt?” (Horace, 20BC) or is there some evidential legitimacy to these fears?

For all the media attention that this subject garners, the supporting scientific literature is still in its nascent stages. The present paper aims to consolidate and integrate some of the key empirical evidence that has emerged regarding the association between smartphone technology and cognitive and affective functioning. We examine the extant corpus of studies in terms of the specific claims put forth by the researchers who conducted them, and where relevant, offer a consideration of factors that might qualify or limit the generalizability of the findings. As we proceed, we evaluate the domains in which there is reason to be concerned about the growing presence of smart technology in our culture, domains in which smartphone technology may enhance cognitive skills, and domains in which the scientific literature is not mature enough to substantiate such claims. In this discussion, we examine evidence relating to both the acute consequences of media technology use on the performance of ongoing cognitive tasks, as well as the more lasting relationships that may exist between technology usage habits and cognitive abilities. As a snapshot of the current literature related to this topic, we also hope this paper can serve as a resource for those conducting further research in this area.

### Challenges and Limitations in Scope

The 21st century has already provided us with a vast array of technological advances that markedly shape the ways by which we interact with the world. In this paper we could not hope to investigate every type of emerging technology, nor would we endeavor to review every psychological implication of the technologies in question. For instance, much has already been written about the impact of violent television and video games on children (Hartmann et al., 2014), and this is one of many topics that fall outside of the scope of the present review. Likewise, this review will not venture into the growing body of research exploring problematic usage of mobile phones and the addiction-like symptoms of overuse (Bianchi and Phillips, 2005; Billieux et al., 2008; Kwon et al., 2013; Lee et al., 2014). Nor will it consider studies exploring the possible effects of radio frequency electromagnetic fields emitted from cellular devices on the human brain and its functioning (Zubko et al., 2016). There is also a growing body of work exploring how technology-related habits may be affecting the development of individuals’ social competencies and emotion reading, and this is yet another topic that has been tackled elsewhere (Brown, 2014; Misra et al., 2014; Uhls et al., 2014; George and Odgers, 2015; Mills, 2016) and to which we give little consideration.

To give the present review some focus, we begin with the premise that smartphones are an especially impactful technological development, due to their flexibility of function, portability, and increasing proliferation. Accordingly, we limit the scope of our examination to work that is directly relevant to smartphone-related impacts. Moreover, rather than concentrating on “problem” behavior related to smartphone technology (see e.g., Bianchi and Phillips, 2005; Hadlington, 2015), we mainly explore evidence regarding the consequences of typical everyday smartphone use. Finally, while a wide array of mental functions might be influenced by smartphone habits, we home in on the impacts in the three domains that are most widely discussed in the lay media and that have garnered some consideration in empirical work: attention, memory, and delay of gratification (reward processing). We then give brief consideration to some emerging work exploring links between smartphone habits, executive functioning, and academic performance.

Some representative studies exploring the relationship between smartphone (and related) habits and cognitive functioning are summarized in **Table 1**. Researchers interested in this area of study are faced with many difficulties when developing an empirical approach, and these challenges necessarily pervade our attempt to review the extant literature. To begin, smartphones have become so ubiquitous that it is nearly impossible to employ true experimental methods with random assignment into different technology exposure/access groups. Even when it is possible to find technology-naïve participants, contrasting them with experienced technology users is likely to be a confounded approach, due to disparities in SES, age, resources, and social expectations among groups who differ in their habits. As a result, much of the literature consists of quasi-experimental and correlational studies, from which strong inferences regarding causality cannot be drawn. The few truly experimental studies that have been performed on this topic typically investigate only momentary effects of smartphone use or deprivation on cognition, rather than long-term impacts.

**Table 1:** Representative publications exploring associations between technology usage and cognitive domains.

Reference	Finding summary
**Attention**	
Alzahabi and Becker, 2013	Frequent media multitaskers are better at task switching; No correlation with dual-task performance
Cain and Mitroff, 2011	Effect of media multitasking on distractor filtering is due to differences in attentional scope rather than working memory capacity
Leiva et al., 2012	Within-phone interruptions cause up to a 4x delay in completion of a primary task
Lui and Wong, 2012	Frequent media multitaskers exhibit better multisensory integration
Moisala et al., 2016	In the presence of distractor stimuli during a sustained attention task, frequent media multitaskers perform worse and exhibit more right prefrontal activity
Ophir et al., 2009	Frequent media multitaskers perform worse on a task-switching paradigm, due to reduced ability to filter out interference
Ralph et al., 2013	Frequent media multitaskers report higher levels of everyday attention failures; No relationship between media multitasking habits and memory failures, attention switching, or distractibility
Ralph et al., 2015	No relationship between habitual media multitasking and sustained-attention processes
Stothart et al., 2015	In an attention-demanding task, mobile phone notifications cause a disruption in performance similar in magnitude to active phone usage
Thornton et al., 2014	The “mere presence” of a cell phone may produce diminished attention and worsened task-performance, especially for tasks with high cognitive demands
Yap and Lim, 2013	Frequent media multitaskers exhibit split visual focal attention, whereas infrequent media multitaskers exhibit unitary visual focal attention
**Memory and knowledge**	
Boari et al., 2012	Forcing users to perform mental rotations, rather than automating them, enhances spatial knowledge acquisition
Burnett and Lee, 2005	Navigation system use impairs cognitive map building
Cain et al., 2016	More frequent media multitasking correlates with poorer working memory performance and lower standardized test scores
Frein et al., 2013	Frequent Facebook users exhibit poorer performance on a free recall task
Henkel, 2013	Taking a digital photograph reduces recall accuracy for details of specific images; This effect is mitigated by zooming in on the object
Parush et al., 2007	The use of navigation systems produces spatial knowledge impairments, but these can be mitigated by requiring users to request their position
Small et al., 2009	Older adults with significant internet experience show increased fMRI activity during internet search relative to those who are ‘net naïve’
Sparrow et al., 2011	When people assume that they have future access to information, they exhibit lower rates of recall for that information, but remember where that information can be accessed
Uncapher et al., 2015	Frequent media multitaskers exhibit poorer working-memory performance and increased attentional impulsivity
Xavier et al., 2014	Internet/Email use predicts better performance on a delayed recall task in the elderly
**Delay of gratification and reward**	
Hadar et al., 2015	Administering smartphones to a smartphone-naïve sample results in greater delay discounting and decreased information-processing ability
Sanbonmatsu et al., 2013	Frequent media multitaskers report greater impulsivity and sensation seeking along with poorer working memory performance
Wang and Tchernev, 2012	Cognitive needs are not satisfied by media multitasking; Emotional gratifications are obtained despite not being sought
Wilmer and Chein, 2016	Greater investment in mobile devices correlates with weaker tendency to delay gratification. This relationship is mediated by impulse control
Zhang and Zhang, 2012	Different patterns of media multitasking result in different sorts of gratification
**Everyday cognition and executive functioning**	
Abramson et al., 2009	More mobile phone usage predicts faster but less accurate Stroop performance
Alloway and Alloway, 2012	Frequent social media users commit more false positives in a Go/No-Go paradigm
Barr et al., 2015	More smartphone usage correlates with more intuitive, less analytic thinking
Baumgartner et al., 2014	Frequent media multitaskers report problems with everyday executive functioning; No relationship between media multitasking and performance on cognitive assessments
Beland and Murphy, 2014	Enforcing mobile phone bans in school is associated with better academic performance
Fox et al., 2009	Instant messaging while reading results in slower reading times, but no difference in comprehension; Higher rates of instant messaging are correlated with lower academic performance
Jacobsen and Forste, 2011	Negative correlation between electronic media usage and academic performance; Positive correlation between media usage and face-to-face interaction
Junco, 2012a	Text messaging and Facebook use during class are negatively correlated with GPA; Email, internet searching, and talking are not correlated with GPA
Junco, 2012b	Facebook use is negatively correlated with GPA; Use for socializing (e.g., status updates), rather than collecting and sharing info (viewing/posting pictures), drives the correlation
Junco and Cotten, 2012	Texting, Facebook, and conducting internet searches unrelated to academic activity concurrent with homework completion all negatively correlate with GPA
Karpinski et al., 2012	Social media use is negatively correlated with academic performance; The correlation is moderated by multitasking habits in a US sample, but not in a European sample
Kirschner and Karpinski, 2010	Facebook use is negatively correlated with GPA and hours per week spent studying
Lepp et al., 2014	Positive correlation between smartphone usage and anxiety; Negative correlation between smartphone usage and academic performance
Levine et al., 2007	Time spent instant messaging correlates with higher rates of distractibility during academic tasks
Mark et al., 2012	Individuals unable to access email for 5 days are less stressed, multitask less, and maintain longer task focus at work
Minear et al., 2013	Frequent media multitaskers exhibit greater impulsivity and lower fluid intelligence; No relationship between media multitasking and task-switching
Paul et al., 2012	Time spent on social networking sites is negatively correlated with everyday attention
Rosen et al., 2013	Accessing Facebook while studying is negatively correlated with GPA
Sana et al., 2013	In-class multitasking with a laptop is negatively correlated with academic performance for the user and all others within sightline of the screen

The majority of studies in this field also employ self-report questionnaires that provide only a narrow window into the relevant behaviors, and that may in some cases provide unreliable indices of the target behavior (Baumgartner et al., 2016). Indeed, the limited evidence we have regarding the compatibility between subjective and objective usage measures indicates that self-report estimates of usage are likely to be of limited reliability, and only modestly correlated (if at all) with actual usage (Andrews et al., 2015). Further, because the landscape of technology usage opportunities is ever-evolving, many of the questionnaires that researchers develop turn out to have a limited “half life,” sometimes becoming dated (or obsolete) before they can be applied more broadly across research labs, or used to establish meaningful longitudinal trends in key behaviors (Roberts et al., 2005; Rideout et al., 2010). Relatedly, the fact that smartphones are a relatively recent development precludes the existence of any broadly generalizable longitudinal evidence. Thus, even when connections between technology and cognition are established, we do not know the extent to which these impacts are lasting. Another crucial challenge is that it can be difficult to assess technology usage habits without intruding on participants’ natural behavior. Attempts to assess smartphone-related habits (questionnaires, diaries, etc.) can draw the participants’ attention to their patterns of use, which could alter their naturalistic behaviors and affect the way in which participants approach laboratory tasks that are meant to assess the cognitive impacts of such habits. In spite of these many challenges, some foundational research has been conducted, and some intriguing patterns are beginning to emerge. In the following sections, we discuss recent research in the areas of attention, memory and knowledge, delay of gratification, and conclude with a consideration of studies investigating more general effects on academic performance and other domains.

## Mobile Technology Use and Attention

A concern that pre-dates smartphone technology is the rising incidence in the diagnosis of attentional difficulties, most specifically ADHD, in children and adolescents (e.g., Visser et al., 2014). Considered together with the rise in the prevalence of multimedia devices, this correlation may be perceived by the public to be evidence of a causative relationship. Opportunities and motives to interact with digital media technologies are especially compelling for today’s adolescents, for whom many social interactions take place online. Such trends have spurred the fear that regular engagement with these devices can lead to diminished attentional capacity – producing shorter attention spans and “scatter-brained” tendencies among those who are most invested with the devices (e.g., Egan, 2016). One specific manifestation of this concern is that the current generation of children and adolescents are developing increasingly shorter attention spans due to their increased contact with smartphone technology, and use onset at younger ages (Nikken and Schols, 2015).

Here we consider the empirical research concerning the potential impacts of smartphone-related technologies on divided attention and focused attention. Focused attention refers to the capacity to attend to only one source of information while ignoring other incoming stimuli. Focused attention also encompasses sustained attention – the ability to maintain a directed attentional focus over an extended period of time. Conversely, divided attention typically refers to the ability to perform two or more functions simultaneously, otherwise known as multitasking.

Perhaps the most recognizable, and obvious, impact of smartphone technology in our everyday lives is the way in which it can acutely interfere with, or interrupt, ongoing mental and physical tasks. It may be useful to think of smartphone-related interruptions as coming in two forms: endogenous or exogenous. Endogenous interruptions occur when the user’s own thoughts drift toward a smartphone-related activity, and thereby evince an otherwise unsolicited drive to begin interacting with the device. These endogenously driven drifts of attention might arise from a desire for more immediate gratification when ongoing goal-directed activities are not perceived as rewarding (Melcher, 2013), a point to which we return below. Once attention has been shifted to the smartphone for one purpose (e.g., by virtue of a specific notification source), users often then engage in a chain of subsequent task-unrelated acts on the smartphone, thereby extending the period of disruption. Studies exploring these ‘within-phone’ interruptions have found that task completion in one app can be delayed by up to 400% by an unintended interruption from another app (Leiva et al., 2012). And, some evidence suggests that the more “rich” (e.g., including a visual image rather than just text) the information encountered during an interruption, the more detrimental the distraction is likely to be with respect to primary task completion (Levy et al., 2016).

Exogenous interruptions occur when some environmental cue captures the user’s attention. This often involves an alert coming directly from the smartphone itself, but can also involve some other external event that triggers subsequent smartphone use, such as noticing someone else interacting with his or her phone, or being reminded during a live conversation (either explicitly or implicitly) about an activity that can be accomplished on one’s smartphone (email, information search, etc.). Importantly, smartphones are capable of interfering with focused attention even when the user attempts to ignore them. In one recently published study, for instance, researchers demonstrated that exposure to smartphone notifications significantly decreased performance on a concurrent attention-based task, even when the participant did not take the time to view the notification (Stothart et al., 2015). Simply hearing the sound or feeling the vibration that signified the alert was enough to distract the participants and decrease their ability to focus attention on the primary task. The researchers posited that that the notifications prompted task-irrelevant thoughts, which manifested themselves in poorer performance on the primary task.

Further evidence suggests that even the mere awareness of the physical presence of a cell phone may impact cognitive performance. Thornton et al. (2014) conducted a study in which participants were asked to complete two neuropsychological tasks designed to measure executive function and attention, a digit cancelation task and a trail-making task. Each task involved two levels of difficulty. At the start of the experiment, the experimenter “accidentally” left either her cell phone or a notebook on the participant’s desk. Participants in the cell phone condition performed significantly worse on the more difficult parts of the digit cancelation and trail-making task than participants in the notebook condition, but performance on the easier parts of the tasks was similar. The researchers replicated these findings in a follow-up study for which half of the participants were asked to place their own cell phones on their desks. The researchers concluded that the mere presence of a phone is sufficiently distracting to affect cognitive functioning, but only during demanding tasks.

Deleterious effects of smartphones on attention are particularly concerning in situations where attention is crucial for safety, such as in the case of distracted driving. A substantial body of work over the past 12 years has considered the effects of texting on driving abilities using driving simulators or closed tracks. Caird et al. (2014) performed a meta-analysis on this literature and concluded that the act of writing text messages impacts nearly every studied measure of dangerous driving. They reported that texting consistently led to decreased attention to the road, slower response time to hazards, greater lateral variance across the lane, and more crashes. Reading text messages without responding resulted in similar findings, albeit with smaller effect sizes. These findings are particularly troubling given that 31% of adults surveyed in 2011, and 42% of teen drivers surveyed in 2015, reported that they had read or sent text messages while driving in the past 30 days (Centers for Disease Control and Prevention, 2011, 2016).

Research investigating the direct impacts that interruptions can have on performance is complemented by research on “resumption errors” – errors that arise in task performance that is resumed following an interruption or task-switch (Monk, 2004; Cades et al., 2007; Brumby et al., 2013). The tendency to commit resumption errors increases steeply when the interruption duration exceeds 15 s (Monk et al., 2008). Smartphone interruptions frequently exceed this 15 s threshold (Leiva et al., 2012), and therefore may be especially deleterious to the resumption of ongoing tasks.

The acute and short-term consequences of having one’s attention distracted away from ongoing tasks is an obvious locus of concern in relation to smartphone habits, but there is also growing fear that the increasingly regular interactions we have with smartphones might also have a more lasting impact on the basic capacity for focused and sustained attention. At this point, very limited empirical evidence lends backing to this concern. Given the lack of longitudinal research in this domain, the best data available are derived from correlational studies. However, findings from those studies are somewhat mixed with respect to the claim that smartphone usage is linked to a diminished attentional capacity beyond the time in which an individual is actively engaged with the device.

One study intimating that smartphone habits diminish sustained attentional abilities was conducted by Lee et al. (2015). The specific focus of their work was on the connection between an individual’s degree of “addiction” to a smartphone and the ability to achieve “flow.” A flow state relates to sustained attention in that it is “a state of concentration so focused that it amounts to absolute absorption in an activity” (Csikszentmihalyi et al., 2014). Lee et al. (2015) investigated whether one’s specific pattern of smartphone usage could have long-term effects on the ability to achieve a state of flow. The researchers administered three questionnaires to a large sample of university students, measuring level of smartphone addiction, tendency for self-regulated learning, and capacity for learning flow. The results showed that the individuals who scored highest on the smartphone addiction scale scored significantly lower on the self-regulated learning and learning flow scales. The authors suggest that the smartphone addiction *causes* a reduced ability to achieve flow and to be self-regulated learners. Of course, it is equally possible that individuals who are able to be self-regulated learners and more easily achieve flow are also more capable of controlling their impulses with respect to smartphone usage, and thus scored lower on the smartphone addiction questionnaire, or that smartphone use and learning flow exert bidirectional influences on one another. Given the correlational nature of the data, we cannot infer any directionality for the relationship, but the data at least hint that excessive smartphone usage could have a negative impact on the ability to maintain the form of sustained focused attention assessed by the flow index.

Prior research on the relationship between smartphone technology and cognitive abilities has also explored a form of media-related divided attention, “media multitasking,” which involves the simultaneous use of more than one media technology, often via a smartphone. Despite the obvious link to work on divided attention, studies exploring media-multitasking are generally not focused on the acute impacts of media engagement on concurrent cognitive activities (e.g., how being on one’s smartphones might affect attentiveness to work activities). Rather, media-multitasking studies mostly explore the associations that exist between one’s basic cognitive skills and one’s tendency to engage in simultaneous media-related habits. In a seminal experiment on this behavior, Ophir et al. (2009) developed and validated the Media Multitasking Index (MMI), a rating determined by responses to a self-report questionnaire (the Media Use Questionnaire) that expressly assesses an individual’s media multitasking habits. They then used computer-based behavioral tasks to measure participants’ attentional functioning. The data revealed that those who reported engaging in more media multitasking were also less able to filter environmental distractions (task stimuli that were inessential to the primary task). Additionally, frequent media multitaskers exhibited higher switch-costs in a task-switching paradigm, indicating that they were less able to suppress the activation of task set representations that were no longer relevant to performance (Monsell, 2003). These data suggest that frequent multitasking of this sort may be associated with a tendency toward allowing bottom-up (environmental) inputs to capture attention (and conversely, a stronger tendency toward exploratory information gathering). Some subsequent studies have replicated and extended aspects of this influential paper. For instance, using a shorter form of the Media Use Questionnaire, Moisala et al. (2016) showed that everyday media multitasking is associated with poorer control over attention. Specifically, the participants who had higher MMI scores made significantly more errors on a task measuring their ability to ignore distractors that interfered with task completion. Moreover, Cain and Mitroff (2011) found that the link between distractibility and media multitasking habits was associated specifically with individual differences in the scope of attention [and not differences in working memory; see also Yap and Lim (2013) for related results].

Brain imaging studies exploring potential neural correlates of habitual media multitasking behavior have demonstrated that the associated attentional deficit may be directly manifest in the functioning of the brain’s attentional control circuitry. For instance, concurrent with the behavioral deficit they observed in performance of a focused attention task, Moisala et al. (2016) showed that individuals with higher MMI scores also exhibited relatively increased activity in right prefrontal areas. The authors interpreted this result as evidence that increased daily multitasking leads individuals to experience greater difficulty in recruiting cognitive control resources. Relatedly, Loh and Kanai (2015) found reduced gray matter in the anterior cingulate cortex of frequent media multitaskers, indicating that this habit may have a direct impact on the structural properties of an important locus of attentional control in the brain (though it should be noted that other functions have also been ascribed to this region; Shenhav et al., 2016).

While these behavioral and neuroimaging findings are intriguing, some research using MMI scores has failed to reproduce the originally observed associations (Minear et al., 2013; Ralph et al., 2013, 2015). Indeed, some evidence suggests the opposite pattern of relationship – that high MMI scores correlate with *better* performance on certain attentionally demanding tasks. For instance, Lui and Wong (2012) created a task that required participants to integrate incoming information from multiple sensory modalities (vision and audition). Their findings revealed that individuals who reported heavier multitasking outperformed light multitaskers in their ability to integrate the information arriving from multiple modalities. Findings suggesting an attentional *benefit* associated with heavier media multitasking are also compatible with studies demonstrating positive and transferable impacts of training, through repetitive task practice, in divided attention tasks (Dux et al., 2009; Karbach and Kray, 2009).

Perhaps because the Media Multiuse Questionnaire was the first questionnaire of its kind to be employed in a study published in a major scientific journal, the measure has been widely adopted as an assessment of media-related behavior, and as such, is the basis of many additional empirical studies. In just the few years since its conception, dozens of studies have used MMI scores to investigate the cognitive and psychological impacts of media multitasking (see **Table 1**).

The use of this questionnaire across laboratories and to explore different dimensions of functioning has provided the field some much needed grounding. The Media Multiuse Questionnaire does, however, have some limitations that might constrain the generalizability of these studies. One potential issue is that the MMI is calculated by submitting subject responses into a formula that applies the same weight to each of 132 potential forms of multitasking (the crossing of 12 different media-related behaviors with any of the 11 remaining behaviors). Thus, one’s multitasking score increases by the same amount regardless of the type of multitasking indicated, and regardless of the relative attentional demands of different media activities (or of combining certain activities with others). For instance, the questionnaire treats the tendency to “Play video games” and to “Listen to music” as equivalent, despite the fact that the former typically requires active attentional engagement and the latter is often a passive pursuit. Likewise, specifying a frequent tendency to “Play video games” while “Reading print media” (books) – a challenging pairing – increases one’s MMI score by the same magnitude as “Listening to music” while “Instant messaging” – a less challenging pairing (in general, this measure may give disproportionately high MMI scores to individuals who frequently listen to music). Relatedly, because the measure includes different listings for “Instant messaging” and “Mobile phone text messaging,” individuals who frequently engage in these activities will have disproportionately high scores because their score is doubly weighted by these now functionally equivalent activities (the consequence of an ever-changing technology landscape). Placing the same mathematical weight on all forms of multitasking included in this index likely muddies the outcomes, making it difficult to distinguish those media multitaskers who engage in the types of difficult pairings [like those used as the basis of training in studies showing beneficial effects of practice with divided attention; e.g., Dux et al. (2009), Karbach and Kray (2009)] from those who are just prone to distracting themselves with secondary sources of input (like music). The limited specificity of the MMI might also account for the recent observation that individuals who fall somewhere in the middle of the media multitasking spectrum may perform better on attentionally demanding tasks than either high or low media multitasking participants (Cardoso-Leite et al., 2014).

While media multitasking appears, at least under certain circumstances, to be negatively correlated with the ability to task-switch and filter distractions, one form of media included on the questionnaire has been associated with *improvements* in multitasking: action video games. As Cardoso-Leite et al. (2015) note, it may seem paradoxical that media multitasking is related to poorer multitasking performance whereas the single task of playing a video game leads to improved multitasking performance. Nonetheless, positive associations between gaming and skills like selective attention, sustained attention, task-switching, and visual short-term memory have been demonstrated in numerous correlational and experimental studies (for a review, see Green and Bavelier, 2012). These associations appear to be specific to the genre known as “action video games” (e.g., first-person shooters), rather than strategy games or role-playing games. Action games require high cognitive and perceptual loads, divided visual processing, and feedback learning with a complex reward schedule, and seem to specifically improve pattern recognition and the metacognitive process of “learning-to-learn” (Green and Bavelier, 2012). The specificity of this relationship highlights another limitation of the MMI: it does not distinguish between different types of video games. Of note, action video games are typically played on computers or gaming consoles, whereas many popular smartphone games (e.g., Candy Crush, Words with Friends) are strategy games that seem to be less likely to confer similar cognitive advantages.

### Attention: Summary

The research reviewed above provides some limited empirical support for claims about the effect of smartphone technology on our attentional capacities. While there is clear evidence that engagement with smart devices can have an acute impact on ongoing cognitive tasks, the evidence on any long-term impacts of smartphone-related habits on attentional functioning is quite thin, and somewhat equivocal. Generally, the evidence does point to a negative relationship between smartphone usage and attention, but correlational and self-report data dominate the literature. Where more controlled assessment of attentional performance has been deployed, such as with media multitasking, the results are mixed, with some studies even yielding a positive relationship with the ability to filter distractions. The limitations of current methods used to measure media-related behavior and wide variation in the specific tasks used to assess attentional performance may account for some mixed results in the literature.

## Mobile Technology Use, Memory, and Knowledge

Smartphones provide constant access to an endless and ever-improving database of collective knowledge. Having this access enables people to search for, locate, and learn seemingly any fact that they desire. Prior to the advent of the World Wide Web, the closest available approximation of this sort of resource was a multi-volume encyclopedia, the cost and limited portability of which precluded ubiquitous use. Internet search engines enable anyone on a connected device to have access to an unfathomably large amount of information, often at very low cost. Moreover, smartphone technology allows people to take this information wherever they wish, and access it within a matter of seconds.

Though it may seem as if constant access to a limitless database of knowledge should improve cognition, much has been written about how the rapidly changing landscape of technology is negatively affecting how we remember our own lives, the places we have been, and those with whom we have interacted (e.g., Kuhn, 2010; Humphreys and Liao, 2011; Pentzold and Sommer, 2011; Frith and Kalin, 2015; Özkul and Humphreys, 2015). However, as with attentional impact, the body of empirical evidence demonstrating tangible effects of mobile media devices on memory and knowledge is limited.

One topic that has been investigated is the oft-cited claim that modern technology is leading us to depend upon our devices to store information for us. In a highly influential and informative study, Sparrow et al. (2011) asked participants to type a series of newly learned trivia facts into a computer. Half of the participants were told that the computer would store their typed information for them and that they would be able to access it later, whereas the other half believed that the information would soon be erased. The individuals who believed they would maintain access to the typed information performed more poorly on a later recall task. Importantly, an explicit instruction to remember the facts vs. not being told to remember had no impact on participants’ rates of recall. This finding, dubbed by the authors as the “Google Effect,” and later referred to by other researchers as “digital amnesia” (Kaspersky Lab, 2015) demonstrates that the expectation of having later access to information can make us less inclined to encode and store that information in long-term memory.

Sparrow et al. (2011) further argued that we are becoming symbiotic with our technology; remembering less actual information and instead committing to memory *where* such information can be found. To further investigate this theory, the researchers conducted an additional experiment using a design similar to that described above, but with three within-subject conditions. For one third of the questions, participants were simply told that the information they entered was saved. Another third of the questions resulted in the participants being told that the information was saved into one of six pre-determined folders (named FACTS, DATA, INFO, NAMES, ITEMS, and POINTS). The remaining third of the questions were followed by a prompt that informed the participants that the information they typed was immediately deleted. The results of this experiment indicated that participants were better able to recall the name of the folder in which the relevant information was located than the information itself. The authors use this finding to claim that, “the processes of human memory are adapting to the advent of new computing and communication technology” (Sparrow et al., 2011, p. 778).

A potential experimental confound that Sparrow et al. do not discuss is the amount of “information” represented by the trivia fact vs. the name of the folder. The authors provide an example fact, “The space shuttle Columbia disintegrated during re-entry over Texas in February 2003.” The complexity of the fact may make it more difficult to memorize than the name of the folder in which the information is stored (i.e., FACTS). Future research should attempt to create more balance between the trivia statements and the folder names.

Barr et al. (2015) recently reported findings from a further exploration of internet access via smartphones and knowledge representation. In keeping with the notion that humans are generally “cognitive misers” (Kahneman, 2011), these authors posited that the tendency to rely on simple heuristics and mental shortcuts extends to the habitual use of internet search engines as a substitute for deep cognitive analysis. In their experiment, Barr et al. (2015) gave participants a series of cognitively demanding questions, including syllogisms, base-rate problems, and a “heuristics and biases” battery. They also assessed participants’ knowledge in different cognitive domains through administration of a numeracy test and a verbal intelligence test. Finally, participants were also asked to provide an estimation of how much time per day they spend on their smartphones overall, as well as an estimation of how much time they spend specifically using internet search engines on their smartphones. The results showed that individuals who reported being heavy users of smartphones also exhibited less analytical “cognitive styles” and poorer performance on the knowledge measures. Moreover, individuals who indicated that they spend a large amount of time using the search engine function on their smartphones scored most poorly on these cognitive measures. Of course, since these results are derived from self-reported data, it is conceivable that participants who highly weight their desire for knowledge may also inflate their memory for (and estimates of) the time they devote to using search engines. Further, given the correlational nature of the research, the results cannot resolve whether, as claimed, frequent search engine use can actually “supplant thinking,” or whether individuals who already have a weaker tendency to engage cognitive analytic strategies also tend to use search engines more frequently [see also Small et al. (2009) and Xavier et al. (2014) for somewhat conflicting findings in older adults].

Interpreted in a different light, Barr et al.’s (2015) results seem counter-intuitive. After all, the tendency to go out of one’s way to seek information and knowledge [e.g., Need for Cognition, (Cacioppo et al., 1984)] has been shown to be positively correlated with fluid intelligence (Fleischhauer et al., 2010). Reinterpreted in this way, individuals with higher cognitive scores might have more semantic knowledge already accessible to them, and thus would not need to resort to using their smartphones as often. Moreover, it is possible that those with higher cognitive scores are able to conduct searches more efficiently. Accordingly, they might use their smartphone’s search engine functions just as frequently as those with low scores, but for a shorter duration each time.

Another recent study provides complementary empirical evidence regarding the potential impact of digital media on memories for personally experienced events (Henkel, 2013). In this study, participants were given digital cameras and taken on a tour of an art museum. Though the research was concerned specifically with digital cameras, the fact that nearly all modern smartphones include a digital camera function makes it relevant to the present discussion. Throughout the tour, the participants were told to take pictures of specific objects, and were asked to observe other objects without taking a picture. One day later, the participants were tested on their ability to distinguish objects they had seen during the tour from brand new objects. The results showed that taking photographs diminished memory for observed objects. Specifically, the participants’ who used the camera during their tour showed a poorer ability to recognize objects as having been previously viewed. A further experiment presented in the same paper showed that this effect was mitigated by asking the participants to zoom in on specific features of the objects that they were viewing before taking the picture. Interestingly, zooming in on a specific area did not increase recall accuracy for details specific to that area vs. the work as a whole, but did improve overall memory for the object, suggesting that the improvement was due to a more rich interaction with the object. Additional empirical support for this phenomenon comes from Zauberman et al. (2015) who found that while visual memory is improved by taking photographs, auditory memory of photographed events is impaired. The practice of taking pictures and videos of trivial occurrences in one’s life (and uploading them to a social media site) is increasingly common due to the proliferation of smartphone ownership and the popularity of photo- and video-sharing social apps like Instagram and Snapchat. If taking pictures can lead to weaker encoding of representations in memory, then this is an important facet of the cognitive impact of ubiquitous smartphone usage. Recent qualitative research provides first-hand accounts that one’s interactions with smartphones and the ‘check-in’ capability of some social media apps as well as photos taken with one’s phone help establish a topographical memory that can both supplant and augment one’s memory of their surroundings and experiences (Özkul and Humphreys, 2015).

Studies investigating the relation between digital photography and memory have assumed that photographs are stored or shared in a semi-permanent matter. Thus, while the act of taking photographs may change memory encoding during an event, the photographs provide an opportunity to review and recollect the experience at a later time. However, recent trends in social media use have prioritized ephemeral photo-sharing. For example, Snapchat – a tool rapidly rising in popularity, especially among youth (Lenhart, 2015) – allows user to send and post pictures and videos that can only be viewed a limited number of times or for a finite period (Instagram recently debuted a similar feature). Users may therefore experience the same effects on memory in the moment, without the added opportunity to refer back to the photograph or video as an external source of information/memory. Little is yet known about the specific effects of ephemeral photo-sharing tools on memory for events (which may act on memory in a way that is akin to the soon-to-be-erased files in Sparrow et al., 2011).

Another common concern regarding the “offloading” of our semantic memory into a modern technological device regards the impact of GPS mapping systems on our ability to navigate the world. Crafting an accurate cognitive representation of our spatial surroundings is crucial for us to effectively and efficiently get from one place to another. It has been posited that constant reliance on GPS navigation systems, which are now integrated into smartphone devices, interferes with our natural tendency to develop cognitive spatial representations. Media headlines insist that these car technologies are “creating stupid drivers” (Moskvitch, 2014) and there are many compelling instances in which a driver blindly followed an inaccurate GPS direction into peril (Hansen, 2013). As GPS navigation devices pre-exist smartphone technology, so too does the related scientific literature.

In a study published a decade ago, researchers sought to identify the consequences of overreliance on GPS navigational devices (Burnett and Lee, 2005). Specifically, the authors wanted to know whether use of GPS navigational devices impacted their participants’ tendency to create cognitive maps when maneuvering through a novel environment. To do this, Burnett and Lee recruited experienced drivers to navigate around a 3D digitally rendered virtual environment. The virtual environment resembled a medium-sized neighborhood, and included many buildings and other landmarks such as trees, signs, and people. The between-subjects design required half of the participants to study a map of the environment for as long as they wished before hitting the road in an attempt to reach their destination using the most direct route possible. Conversely, the other half of participants were allowed to study the map for only 20 s, and then commenced their journey, which was accompanied with turn-by-turn voice guidance to the destination. After the participants completed the route, their spatial knowledge of the environment was tested according to three facets of spatial representation: Landmark-, Route-, and Survey-level representations. Participants were presented with screen shots of scenes, including some from the virtual environment and some that were similar, but not actually on the route that the participants took. The participants were required to identify which screenshots they recognized as part of the route they took (Landmark) and the order in which they occurred (Route). To assess Survey knowledge of the spatial environment, participants were asked to sketch a map of their overall route as best they could on a blank sheet of paper, and to include as many landmarks as they could remember. The results from this study showed that the participants in the voice navigation group performed significantly worse in Landmark and Route knowledge of the environment. Further, those in the voice navigation group drew significantly simpler and more fragmented maps in the assessment of Survey knowledge.

Some recent research has focused on identifying ways in which the detriments of navigation devices on spatial memory can be mitigated. It has been shown, for example, that spatial knowledge can be improved by allowing users to request that their position be indicated at any given time during the navigation episode (Parush et al., 2007). Further, spatial knowledge can be improved if users are forced to perform mental rotations of on-screen images, as opposed to observing automated rotations (Boari et al., 2012). This knowledge can be applied by encouraging users to keep their navigation devices set such that North is always facing up, rather than moving around the compass as they turn.

Finally, research extending the Ophir et al. (2009) findings on media multitasking also implicates this behavior in memory functioning. Most recently, Uncapher et al. (2015) showed that frequent media multitaskers differed from light users with respect to their working memory capacity, and also exhibited diminished long-term memory functioning. In their study, frequency of media multitasking specifically predicted how participants encoded information, with higher rates of media multitasking leading to less precise representations of goal-relevant information and more task-irrelevant information filling the space. Further, the reduced precision of information in working memory observed in heavy media multitaskers was associated with diminished long-term memory performance, as measured by a surprise recognition test for tested items [with a significant association between heavy media multitasking and memory for target items in the earlier working memory task, as well as a trend level association for memory of distractor items; see also Frein et al. (2013) for related findings].

### Memory and Knowledge: Summary

Research investigating the relationships between smartphone technology habits and one’s memory and knowledge capabilities is still scant, but available findings indicate that, as some have worried, smartphone-related habits can in some cases be detrimental to mnemonic functioning. Though there are some important limitations in the experimental designs that have been discussed, the work conducted to date does give us reason to be cautious about how we use new technologies. The available evidence suggests that when we turn to these devices, we generally learn and remember less from our experiences. While the research discussed in this section represents an important step toward investigating the impact of smartphone technology on memory, it is equally important to bear in mind that the sort of “memory externalization” that these articles focus on is by no means a new issue. The same concerns could, for instance, be made regarding a Rolodex. Invented in the 1950s, this ‘rolling index’ provided a system to organize one’s contacts into an easy to access alphabetized structure. It allowed its users to remember *where* an individual’s contact was located, rather than needing to memorize the full contact information. Determining whether externalizing cognitive processes via smartphone is necessarily worse than externalizing cognitive processes via older methods will be an important avenue for future research.

## Mobile Technology Use, Delay of Gratification, and Reward Processing

In addition to their effects on memory and attention, smartphones and related media are often implicated as the cause of a perceived cultural shift toward a necessity for immediate gratification (Alsop, 2014). Indeed, there is a common belief that the current generation of children and teenagers are less capable of waiting for rewards, due in part to the omnipresence of various types of multimedia in their lives (Richtel, 2010b). As with the previous sections, the empirical work exploring this claim is still in its nascent stages. In this section, we outline some studies that inform our understanding of the potential impacts that smartphones can have on individuals’ tendencies to choose smaller, more immediate, rewards over larger rewards after a delay, and then offer a summary on the status of the claim.

Some work in this realm has begun by exploring the motivations that drive individuals to engage with media in the first place. In one such study, Wang and Tchernev (2012) investigated media multitasking in terms of the Uses and Gratifications theory (Katz et al., 1973). Based on this theory, “Needs” could be defined as “the combined product of psychological dispositions, sociological factors, and environmental conditions that motivate media consumption” and “Gratifications” as the “perceived fulfillment” of those needs, in this case as a result of media use or exposure (p. 495). In their experiment, Wang and Tchernev (2012) collected self-reported data over a period of 4 weeks. Participants were asked to submit three reports daily, in which they indicated the types of media that they had used in the time that had passed since the previous report, and whether they performed any of these activities simultaneously (i.e., multitasking). The participants were also asked to indicate the specific “motivation” (emotional, cognitive, social, or habitual) that drove them to engage in each media interaction, and the strength of that motivation on a 1–10 scale. The participants indicated the degree to which each “need” was satisfied on a 1–4 scale, and this data was aggregated into “gratification” measures used in data analysis. By comparing the various types and strengths of motivations and gratifications across time points, the experimenters were able to draw interesting conclusions regarding the short-term causes and effects of multimedia interaction. Specifically, participants most often reported that “cognitive” motivations drove their interactions with media devices. However, subjective reports indicated that the ensuing interaction with a media device rarely satisfied the cognitive needs. Instead, participants experienced an *emotional* gratification that they did not report pursuing in the first place. Ultimately, these emotional gratifications may be driving subsequent media interactions at an unconscious level [for related findings, see Zhang and Zhang (2012)].

In a study performed in our own lab (Wilmer and Chein, 2016), we used a measure of self-reported mobile technology usage in an attempt to mine the potential relationship with delay of gratification. We observed a significant negative correlation between participants’ mobile technology usage and their “indifference point” and discounting rate in a delay discounting paradigm. Specifically, individuals who were heavier users of mobile technology were also more apt to accept a smaller, more immediate reward than to wait for a more substantial but delayed reward. These findings fit with the popular conception that having constant access to these devices could generate a need for instant gratification. In our study we further observed that the correlation between technology habits and delay of gratification was mediated by individual differences in impulsivity, but not in reward/sensation seeking. This finding partially replicated earlier investigations of the relationship between media use and impulsivity (Minear et al., 2013; Sanbonmatsu et al., 2013; Shih, 2013). Since the results from all of these studies are entirely correlational, they could simply reveal that people who naturally tend toward more immediate gratification and who give in to impulses more easily also tend to use their mobile devices more often (i.e., there may not be a causal relationship from media use to discounting behavior).

Still, habituating oneself to constant immediate gratification could have significant and lasting cognitive consequences. In one of the few truly experimental studies in the field, researchers sought to determine whether non-users of smartphones would exhibit a change in their reward processing capacity after being provided with a smartphone for the first time (Hadar et al., 2015). The aim of the study was to investigate the cognitive, behavioral, and neural consequences of smartphone usage, with a specific emphasis on delay discounting. Participants were divided into three groups: heavy smartphone users, smartphone non-users, and a third group which were smartphone non-users who were given a smartphone for the first time (the latter two groups were assigned randomly). The heavy smartphone users showed higher scores for impulsivity and hyperactivity on a questionnaire that was administered at the beginning of the experiment. Even more interestingly, after a 3-month exposure to smartphones, the non-users who were given a smartphone were found to have become more immediacy oriented in the delay discounting measure, whereas non-users’ orientation did not change. The data from Hadar et al. (2015) suggest that heavy smartphone usage can causally reduce an individual’s capacity (or at least tendency) to delay gratification in favor of a greater reward in the future. These findings are strengthened by the study’s experimental design.

Evidence from neuroimaging research suggests that that neural circuitry implicated in reward processing also plays a role in activities performed on mobile phones, particularly social media. For example, Sherman et al. (2016) found that receiving many “Likes” on one’s social media photographs is related to increased activation in the brain’s reward circuitry, including areas in the dorsal and ventral striatum and ventral tegmental area. The ventral striatum has also been implicated in the experience of sharing information about oneself with peers, a popular activity on social media (Tamir and Mitchell, 2012), and level of response in this brain region has been shown to correlate with level of social media use (Meshi et al., 2013).

### Delay of Gratification and Reward: Summary

As with the research highlighted in the previous sections of this paper, the data is still too sparse to support firm conclusions regarding the impacts of smartphone use on reward processing and delay of gratification. Lurid claims that smart devices are “rewiring our brains” (Greenfield, 2013) into being addicted to instant gratification suffer from a lack of any longitudinal evidence, and still very limited empirical support of any kind. Future research could use neuroimaging techniques to investigate whether any “rewiring” is actually occurring, and some relevant work is currently being conducted. At present, neuroimaging research has been limited to cross-sectional studies mapping the neural correlates of engaging in popular activities on mobile phones. These studies cannot shed light on the ways that mobile phones may be leading to functional or structural changes in the brain. By conducting brain scans before and after a long-term intense exposure to electronic immediate gratification, neuroscientists could analyze whether any connectivity changes occurred.

## Mobile Technology Use and Everyday Cognitive Functioning

Given the pattern of findings in attention, memory, and the ability to regulate reward-related processing in the context of delay of gratification, it follows that we might expect to see links to more generalized measures of cognitive functioning. One way in which such links have been studied is by exploring the relationship between technology habits and general academic performance. Studies on this front generally support the conclusion that poor academic performance (generally assessed by GPA) can be predicted by higher levels of smartphone use (Beland and Murphy, 2014; Lepp et al., 2014), instant messaging (Levine et al., 2007; Fox et al., 2009), social networking (Kirschner and Karpinski, 2010; Junco, 2012b; Karpinski et al., 2012; Paul et al., 2012), media multitasking (Junco, 2012a; Rosen et al., 2013; Sana et al., 2013), and general electronic media usage (Jacobsen and Forste, 2011; Junco and Cotten, 2012).

Researchers have also directly investigated the relationship between mobile technology/media multitasking habits and executive functions that are thought to be essential to academic performance (Abramson et al., 2009; Alloway and Alloway, 2012; Alzahabi and Becker, 2013; Lepp et al., 2014; Barr et al., 2015). In one relevant study (Baumgartner et al., 2014), participants were required to complete a self-report questionnaire and computerized tasks that assessed executive functions in three subcategories: working memory, inhibition, and shifting. Participants who reported being high multitaskers, based on MMI score, also self-reported lower levels of “executive function in everyday life” on the questionnaire. The correlation was significant for all three subcategories of the executive functioning questionnaire. Though the results from the self-report measures were not corroborated by any of the performance-based measures of executive functioning used in that study, very recently published work conducted by Cain et al. (2016) does provide evidence for such links. These authors found that higher media-multitasking among a large adolescent sample was associated with poorer performance on one laboratory measure of executive function, the n-back working memory task, and also with lower scores on a standardized test of academic achievement in the classroom. Taken together, this body of work suggests that the degree to which one can exert executive control over behavior and maintain goal-related representations (in working memory) may explain individual differences in vulnerability to the “real life” consequences of mobile device habits.

Interestingly, there is also some evidence suggesting that one’s susceptibility to cognitive disruption from mobile technology use, and the consequent impacts on academic success, might depend on the individual’s existing cognitive skill set; especially their ability to exert self-regulatory control over behavior. Research indicates, for instance, that how closely an individual monitors and plans for interruptions, via executive control, mediates the relationship between multimedia interruptions and resultant stress (Tams et al., 2015), and that differences in working memory capacity (which is closely linked to executive functioning) is a predictor of the speed of task resumption following an interruption (Werner et al., 2011).

As a further point, it should be acknowledged that some of the cognitive and affective consequences of smartphone/technology habits may come from indirect impacts, such as through influences on sleep and mood. Quality of sleep has been shown to have a serious effect on cognitive performance (Lim and Dinges, 2008), and considerable evidence implicates smartphone technology as a source of sleep disturbances (Cain and Gradisar, 2010 for a review), with a compound effect on cognitive functioning and work engagement the following day (Lanaj et al., 2014). An observation that predates the emergence of smartphone technology is that using electronic devices with a brightly lit screen immediately before bed, such as a television or a computer, can negatively impact one’s ability to fall asleep. Smartphones potentially exacerbate this problem because people frequently keep and charge smartphones at their bedside, often using them as an alarm clock. Based on a recent survey, over 70% of Americans follow this behavioral pattern (Trends in Consumer Mobility Report, 2015). Moreover, in addition to the bright light, it has been proposed that specific activities, such as social interactions and games, occurring via one’s smartphone can lead to psychological arousal and stimulation that could further disrupt subsequent sleep (Cain and Gradisar, 2010). Though most studies in this domain have had child and adolescent participants, recent research has affirmed that this effect can be seen in older adults as well (Exelmans and Van den Bulck, 2016). Future research should investigate a direct relationship between habitual smartphone usage before bedtime and cognitive abilities. Furthermore, future research might attempt to identify if particular smartphone activities (e.g., gaming, passive or active social media use) are especially deleterious to sleep quality, and how notification settings may impact sleep disruption, and also consider how sleep-tracking apps (e.g., the recently introduced “Bedtime” feature on the iPhone operating system) might improve quantity or consistency of sleep.

Extending this work on sleep, Lemola et al. (2014) used self-report questionnaires to explore how sleep and smartphone habits might also impact mood; specifically depressive symptoms. They found that difficulty sleeping was a significant mediator in the relationship between electronic media use and depressive symptoms. While psychopathological symptoms are not the focus of this paper, it is noteworthy that depression is often comorbid with cognitive disorders (American Psychiatric Association, 2013), and that sleep quality is inversely related with cognitive performance (Lim and Dinges, 2008).

Like depression, anxiety is known to have significant negative effects on several aspects of cognitive functioning (American Psychiatric Association, 2013). In early work linking anxiety symptoms, technology habits, and cognitive functioning, Mark et al. (2012) found that limiting individuals’ access to email reduced anxiety and improved later focus on work related tasks. A commonly repeated assertion regarding today’s digital world is that people feel a ‘need’ for access to their phones. Researchers have gone so far as to refer to this phenomenon as a “phantom limb” (Turkle, 2011). Similarly, “phantom vibration syndrome” describes a commonly experienced phenomenon in which people perceive a vibration in their pocket, when no such vibration occurred (Rosen, 2013), and even when their phone is not in their pocket (for a review, see Deb, 2014). In acknowledging the strength of individuals’ attachment to their smartphones, researchers have begun to investigate the degree to which separation from one’s smart device can cause symptoms of anxiety. In one study (Cheever et al., 2014), participants were randomly assigned to one of two groups. One group kept their phones with them for the entirety of the study with the ringer silenced and vibration turned off, whereas the second group had their phones removed from them for the duration of the study. The participants then completed a series of three anxiety assessments at 20-min intervals, followed by a wireless media device usage questionnaire. The researchers posited that the group that had their phones removed from them would experience significantly more anxiety than those who were allowed to keep their phones with them. Although this main effect was not observed, the researchers found that the group of participants who did not have their phones with them scored higher on each successive anxiety test, showing that their anxiety increased as a function of time without their phones. Additionally, the study found that individuals who scored higher on the wireless media device usage questionnaire had higher rates of increased anxiety for the later tests, regardless of whether their phone was taken from them or with them but silenced. These findings led the researchers to conclude that one’s regular mobile device usage predicts the levels of anxiety that results from being separated from their device. However, it is also important to bear in mind that the directionality of the effect remains ambiguous, with research also suggesting that life-stress is predictive of mobile device usage, driven by the social support one can attain through using one’s device (Chiu, 2014).

Clayton et al. (2015) also investigated the impact of brief separation from a mobile device, but additionally assessed the potential impacts on cognition. The researchers focused exclusively on iPhone users, based on the ease with which one can toggle the iPhone’s ringer. Upon arrival, participants were randomly assigned into one of two groups: one group completed a task that is sometimes used to measure sustained attention (a word-search task), first with their phones in their possession and then with their phones given to the experimenters, whereas these conditions were reversed in the second group. Physiological and self-report measures were used to track anxiety levels throughout the experiment. In this experiment, the researchers did more than simply separate the phone from its user; the phone was placed in an adjacent cubicle, and the experimenters placed a call to the phone so that it emitted a ring that the participants’ presumably recognized as their own. The results showed that participants’ anxiety levels were highest when they were separated from their ringing phones and lowest when their phones were in their possession. Moreover, the participants’ performance on the word-search puzzles was significantly poorer when they were separated from their ringing phone.

The data provided by such experiments offer evidence of the psychological sway our digital lives can hold over us. Yet, there is nothing to indicate whether the resultant anxiety is specific to separation from one’s smartphone, or whether the same effect might emerge when participants are separated from something else of subjective value, such as a wallet or personally cherished item. Moreover, the potential implications with respect to cognitive functioning are still limited in that a link between anxiety and cognition was established only via a word-search puzzle, a task that is somewhat idiosyncratic relative to tasks used more typically in cognitive research. The design also does not allow for a determination of whether the effect on word search performance was caused by the absence of the participants’ phones or simply by the distraction of the ring.

## Conclusion

Smartphones (and related mobile technologies) have the potential to affect a wide range of cognitive domains, but empirical research on the cognitive impacts of smartphone technology is still quite limited. This is understandable, given that the relevant technology itself is still young and constantly evolving. However, with each passing year, smartphones become more omnipresent in our lives. Rather than applying to only a niche group of individuals, the research conducted in this domain will soon be relevant to the majority of the world’s population (eMarketer, 2014). Therefore, it is crucial to understand how smartphone technology affects us so that we can take the steps necessary to mitigate the potential negative consequences.

Although the research concerning the potential cognitive impacts of smartphone technology is growing, the results remain contradictory and inconclusive. The at times contradictory findings suggest that not all smartphone use is created equal; certain apps, approaches to multitasking, or notification settings may moderate the relation between overall smartphone use and various cognitive skills. Despite the inconclusive nature of the literature, media headlines encourage a public perception that the findings are conclusive and that smartphones have a definite and negative impact on cognitive functioning. A common view, that smartphones are stifling our creativity by depriving our brains of downtime (Richtel, 2010a), even led to a radio challenge, in which thousands of people reduced their smartphone usage in an attempt to increase their creativity (Zomorodi, 2015). However, there is no extant research to validate the basic concern that motivated the challenge. Investigating the cognitive impacts of filling the small breaks in our day with inputs from smartphone engagement is perhaps another endeavor worth pursuing, but not one that is yet represented in the peer reviewed literature.

As discussed earlier in our review, there are many limitations to the literature that forms the basis for this paper. Chief among these is that there is very little longitudinal evidence on the long-term consequences of frequent smartphone usage. Now is the time to begin gathering the data for such studies. A particularly important topic that requires longitudinal data is the effect of smartphone ownership on young children. Despite widely publicized recommendations (AAP Council on Communications and Media, 2016a,b), we know very little about the most appropriate age for a child to begin using a smartphone, and we know equally little about the consequences of using one too early in life. A longitudinal study with a large sample size should be developed in which children are assessed on a variety of cognitive (and affective) outcome measures at multiple time points. In a study such as this, data could also be gathered to ascertain the degree to which children with smartphones or other portable sources of immediate gratification, such as portable video game systems, are influenced by these devices. Analysis of group differences in rates of maturity of certain cognitive processes could also provide information about how smartphone technology can affect the brain during periods of heightened developmental plasticity. It is possible, but untested, that frequent smartphone usage could be less harmful to adults, whereas children may experience more negative consequences as a result of their increased neural plasticity.

If emerging research does suggest that there are serious consequences of smartphone usage, we need to investigate potential practical approaches that could mitigate these effects. Finally, the majority of the literature only speaks broadly about “smartphone usage.” Future research should distinguish between specific types of smartphone usage, each of which are likely to have differential effects on the user. In particular, it seems likely that social activities such as text messaging, email, and social media use will have different impacts than gaming or browsing the web, yet very little is known about the specific concerns related to these seemingly disparate patterns of use.

As smartphones have worked their way into the pockets of over 70% of American adults, and nearly 50% of adults worldwide, there is also a great opportunity to use them as a tool for research (Poushter, 2016). Scientists have already begun to suggest that smartphones could present a more convenient and more naturalistic method of gathering empirical data for cognitive and social psychology experiments (Raento et al., 2009; Dufau et al., 2011; Miller, 2012). Moreover, as smartphones become increasingly interlaced with our cognitive functioning, it will be important to continue to gather detailed usage metrics to understand how these interactions are affecting us, and how are lives are accordingly shaped.

The research outlined in this paper lays a foundation on which a seemingly endless number of “next steps” can be imagined. There is an immense opportunity for additional research to be performed with the aim of giving psychologists and the world-at-large a better understanding the short-term and long-term effects of smartphone technology.

## Author Contributions

HW and JC conceived and developed the project. HW conducted primary literature review with additional input from LS, both working under the supervision and guidance of JC. HW wrote the initial draft manuscript. JC provided primary editorial feedback, and both JC and LS contributed additional written sections to the manuscript. All authors were involved in final preparation of the manuscript.

## Conflict of Interest Statement

The authors declare that the research was conducted in the absence of any commercial or financial relationships that could be construed as a potential conflict of interest.
